# Supplementary material for: PCAT: an integrated portal for genomic and preclinical testing data of pediatric cancer patient-derived xenograft models
Source: Nucleic Acids Res. 2020 Aug 18;49(D1):D1321–7. doi: 10.1093/nar/gkaa698 (PMC7778893; doi:10.1093/nar/gkaa698)
Supplement: gkaa698_Supplemental_Files [file gkaa698_supplemental_files.zip › Supplementary Figures.pdf]

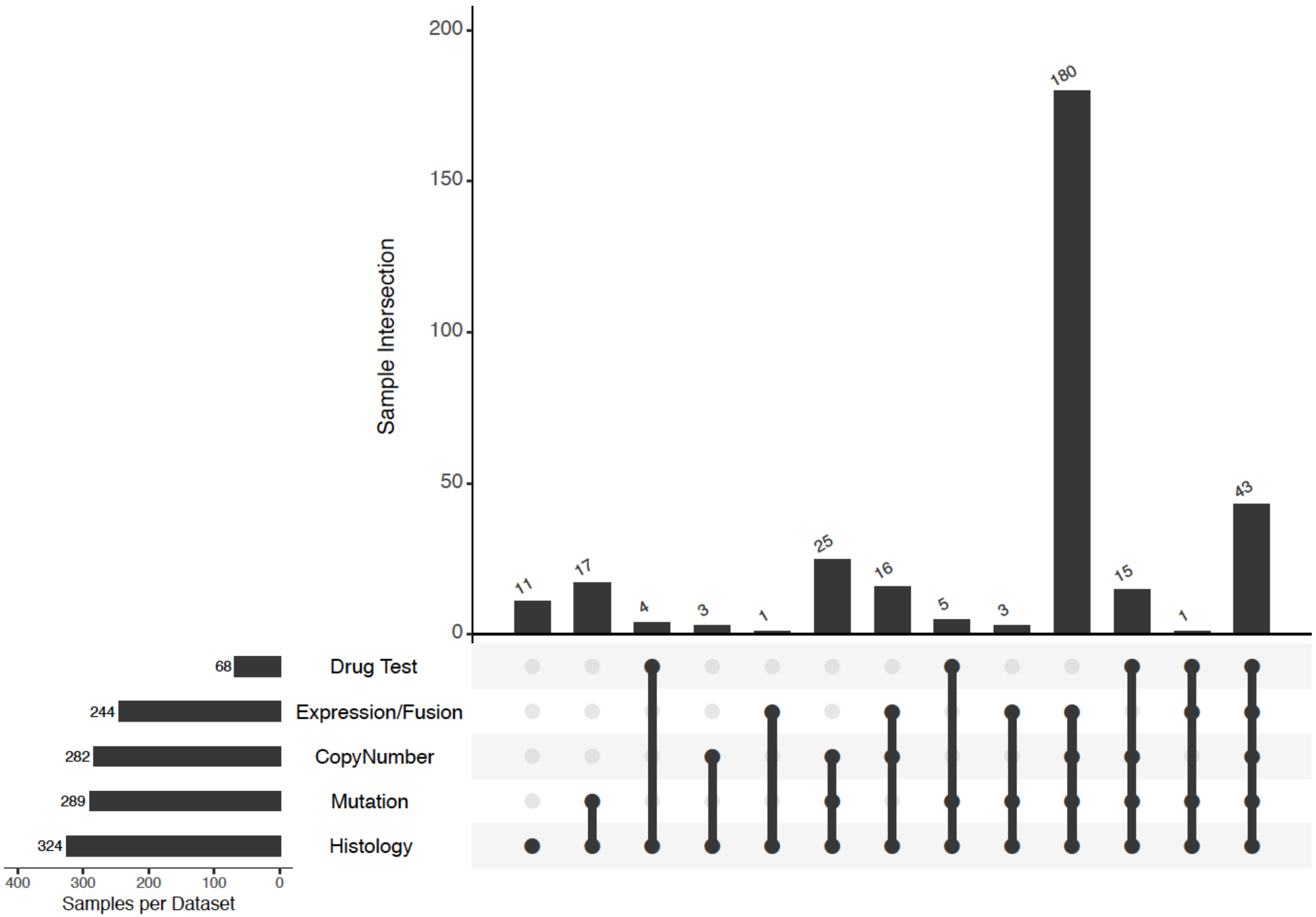

Supplementary Figure 1. A summary of PDX data currently hosted at PCAT. The bars on the left show the total number of samples for each data type. Bars on top show the number of samples with the overlapping data indicated by the solid connecting dots.

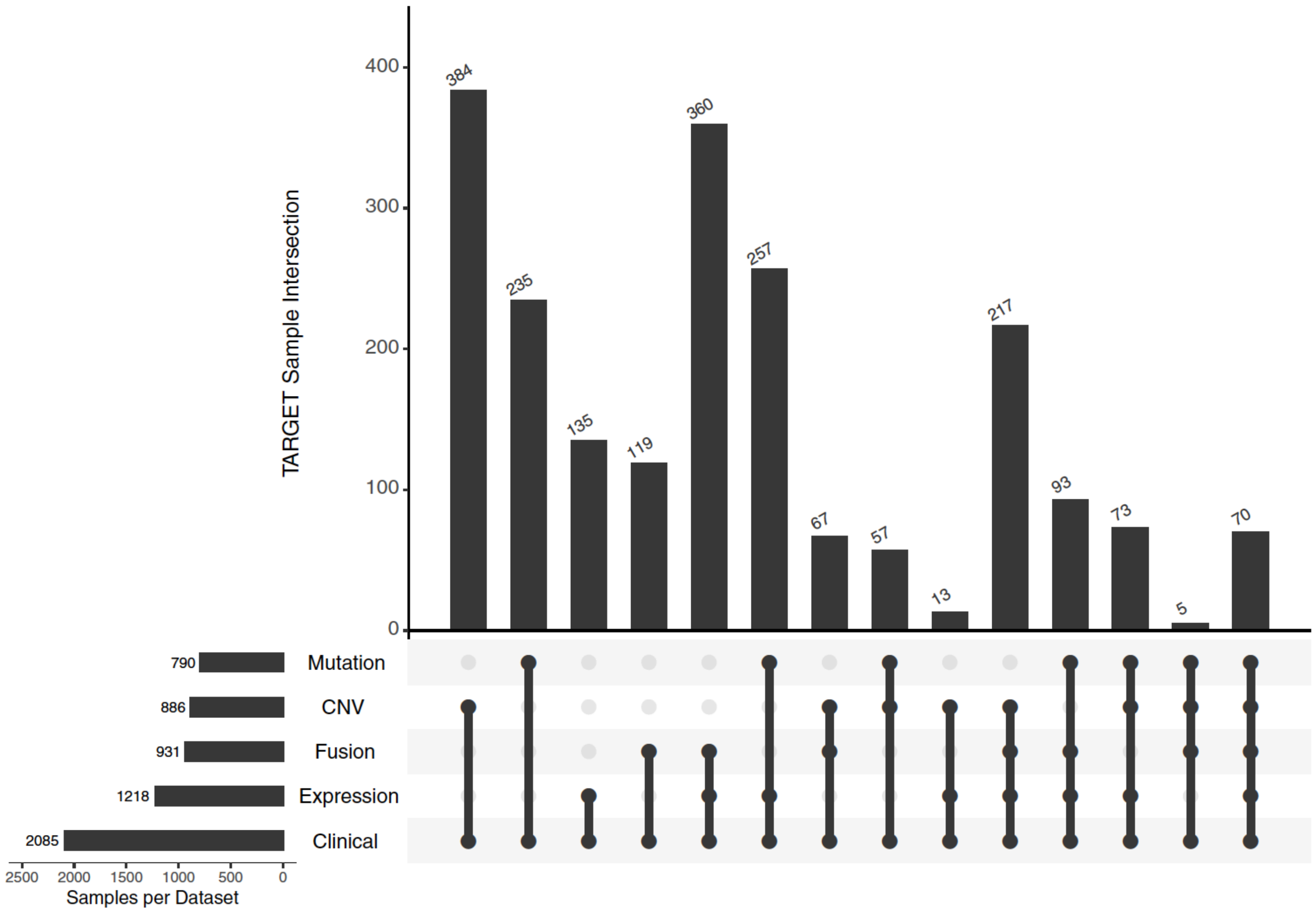

Supplementary Figure 2. A summary of the TARGET data that have been integrated into PCAT. The bars on the left show the total number of samples for each data type. Bars on top show the number of samples with the overlapping data indicated by the solid connecting dots.

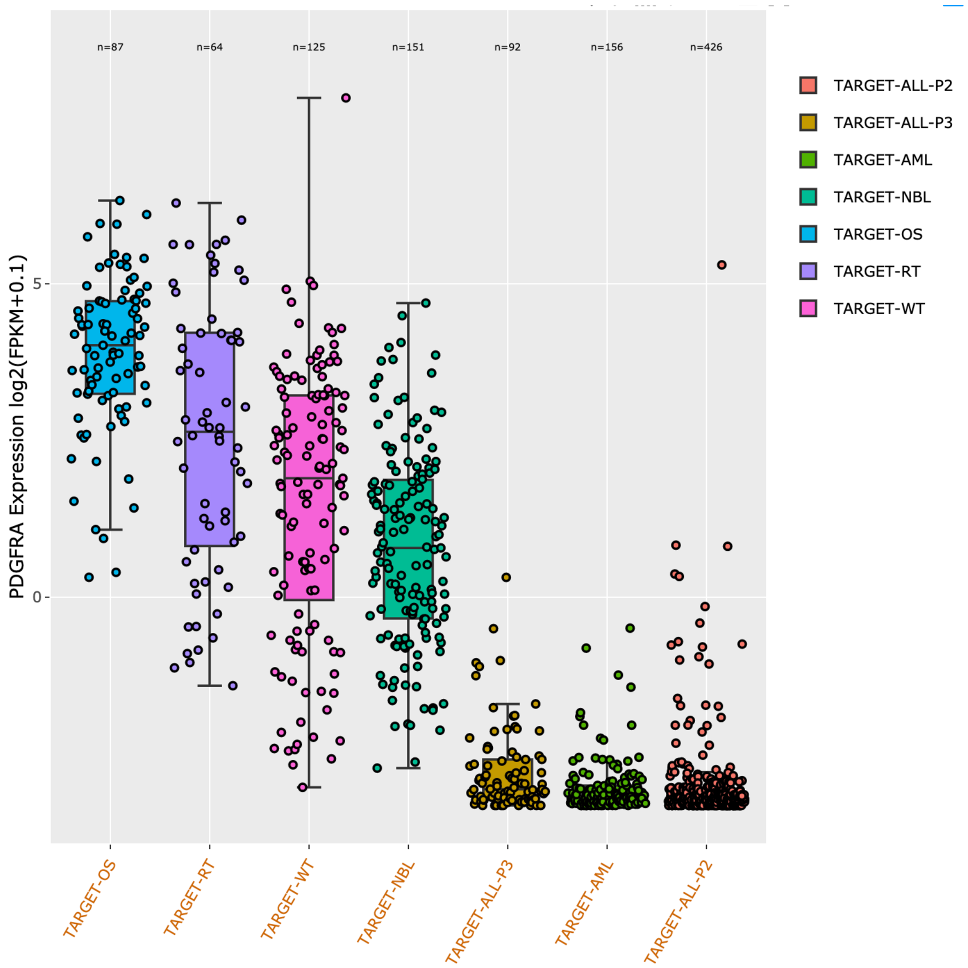

Supplementary Figure 3. PDGFRA expression across TARGET cohorts. Similar to observations made in PDX models, PDGFRA is highly expressed in cancers of mesenchymal origin including osteosarcoma and rhabdoid cancer but very low in blood cancers.

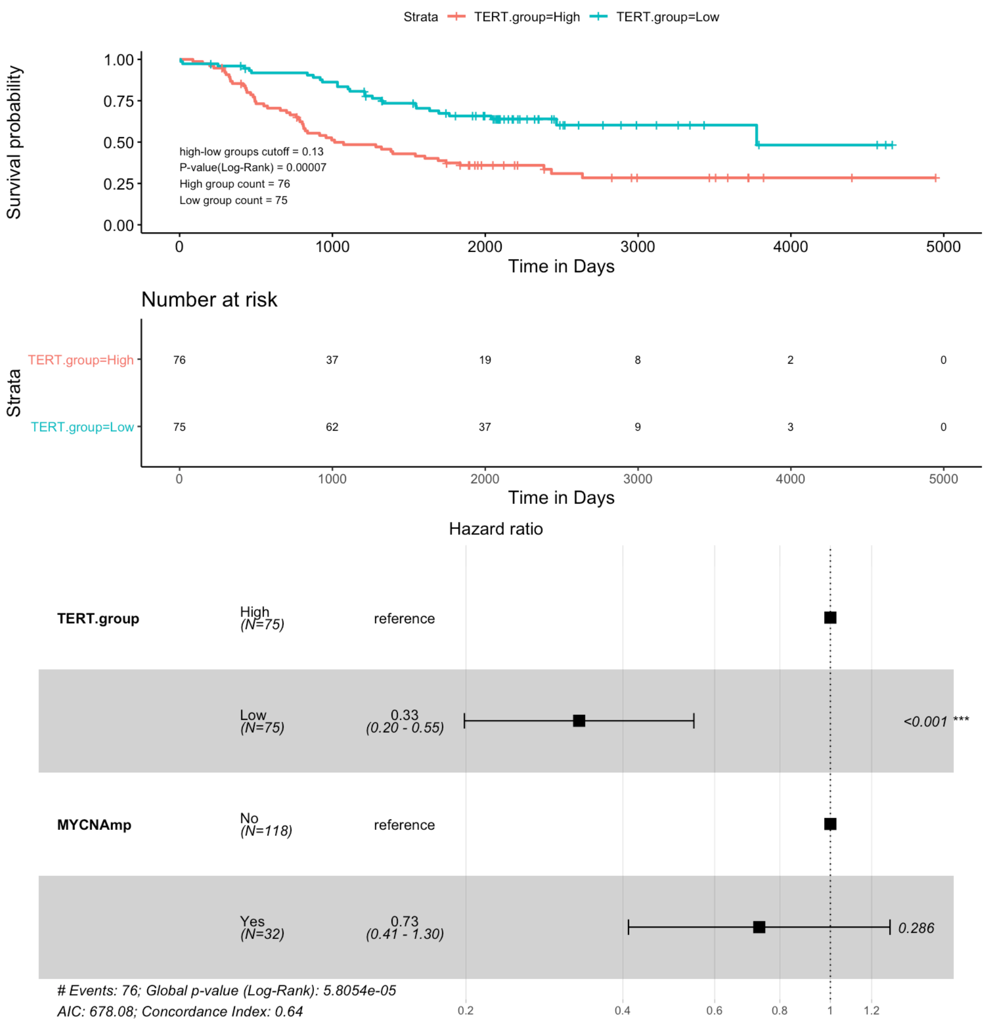

Supplementary Figure 4. Multivariate survival analysis. This example tests the correlation between TERT expression and neuroblastoma prognosis. MYCN amplification status is included to demonstrate that the prognostic value of TERT expression is independent of MYCN.

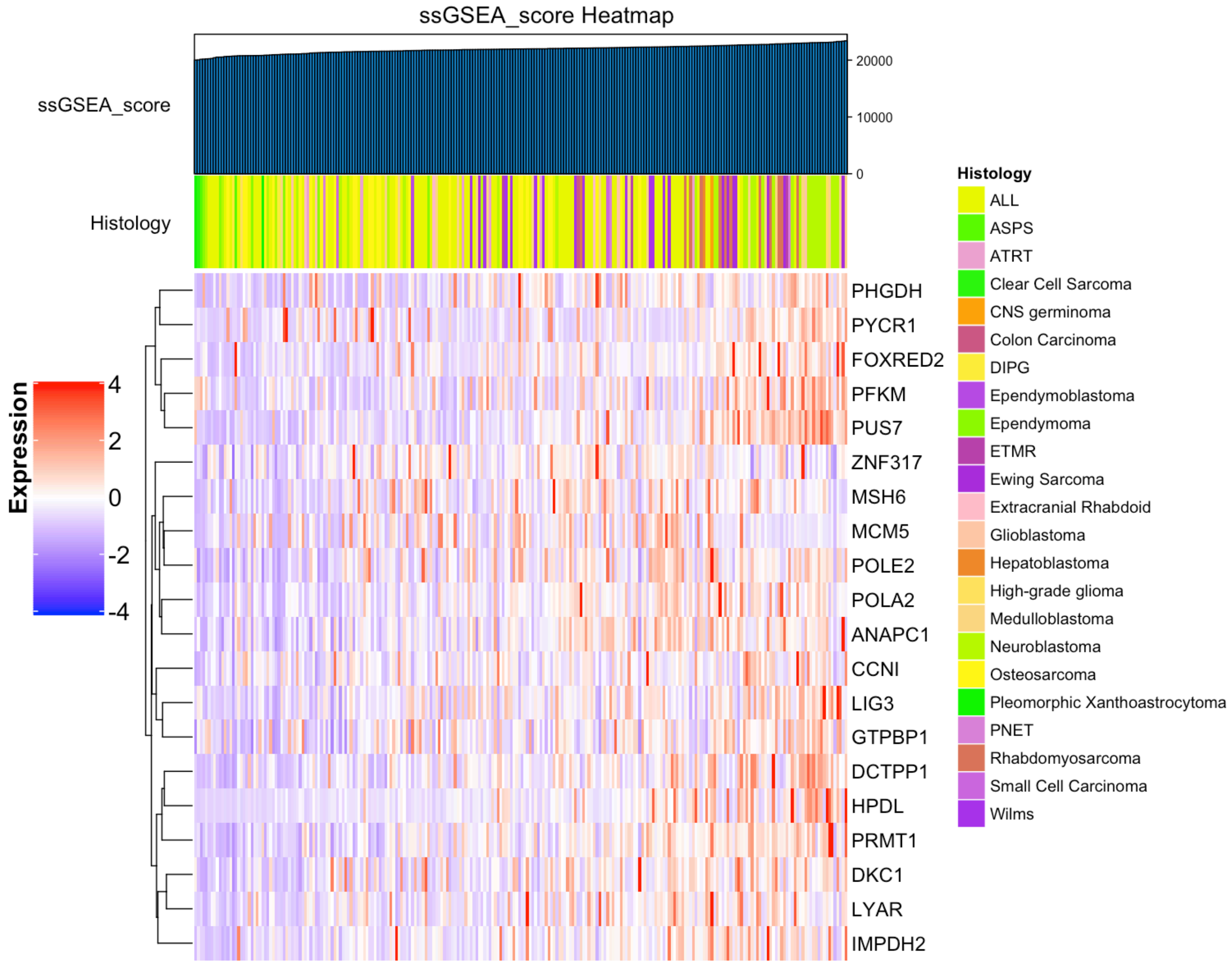

Supplementary Figure 5. ssGSEA analysis of MYCN targets in PDXs. The top 20 genes positively regulated by MYCN were downloaded from Valentijn et al. PNAS 2012. These genes were identified from shRNA screening in the original study. Each column represents one PDX model. Samples are odered from low to high based on ssGSEA scores.

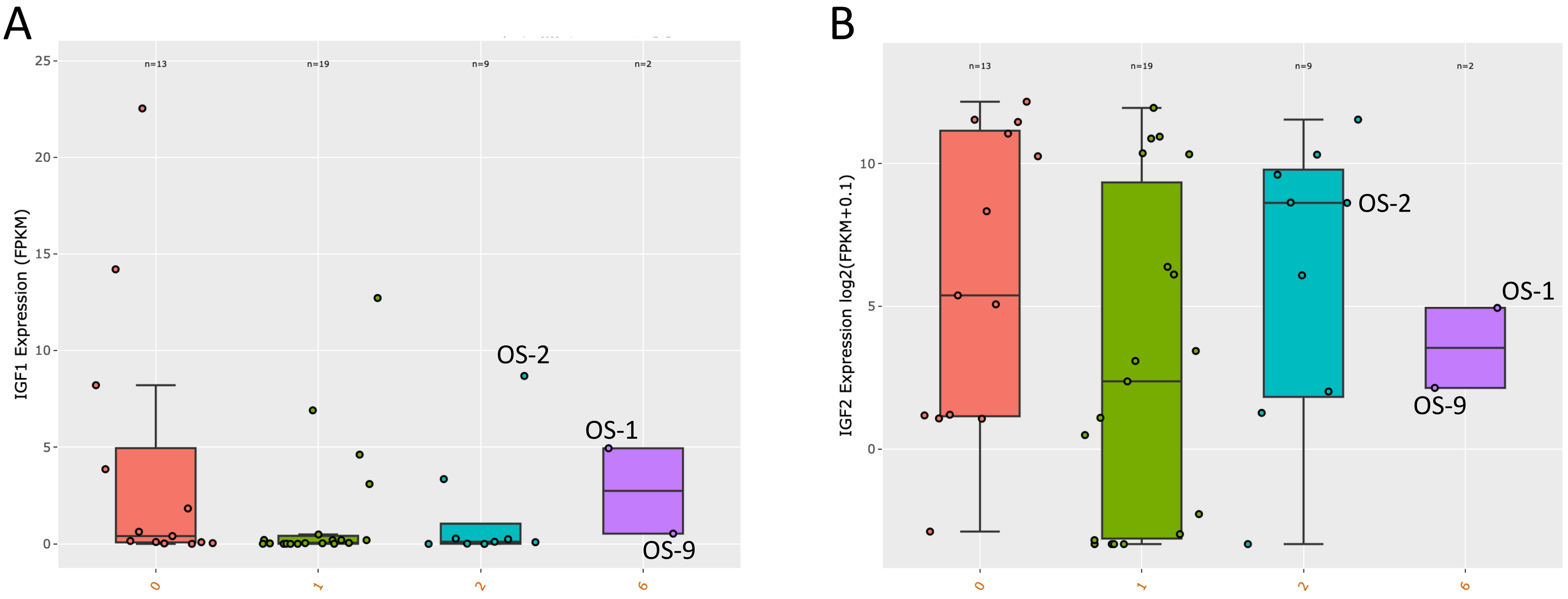

Supplementary Figure 6. Molecular correlates of drug responses. (A) Although OS-1 and OS-9 show MCR to IGF1R inhibitor 19D12, another osteosarcoma model OS-2 shows no response (progressive disease) despite higher IGF1R expression. This figure shows OS-2 also has higher expression of IGF1 than the two responsive models. X axis reflects response levels: 1-2, progressive disease; 6, maintained complete response. (B) OS-2 has higher IGF2 expression than OS-1 and OS-9. Note that in this figure the y axis is in log scale.
